# Antenatal Glucocorticoid Exposure Results in Sex-Specific and Transgenerational Changes in Prefrontal Cortex Gene Transcription that Relate to Behavioural Outcomes

**DOI:** 10.1038/s41598-018-37088-3

**Published:** 2019-01-24

**Authors:** Andrea Constantinof, Vasilis G. Moisiadis, Alisa Kostaki, Moshe Szyf, Stephen G. Matthews

**Affiliations:** 1 Departments of Physiology, University of Toronto, Toronto, ON M5S1A8 Canada; 2 Departments of Pharmacology & Therapeutics, Sackler Program for Epigenetics & Psychobiology, McGill University, Montreal, QC H3G1Y6 Canada; 3 Departments of Obstetrics and Gynecology, University of Toronto, Toronto, ON M5S1A8 Canada; 4 Departments of Medicine, University of Toronto, Toronto, ON M5S1A8 Canada; 5 Lunenfeld-Tanenbaum Research Institute, Sinai Health System, Toronto, ON M5G1X5 Canada

## Abstract

Synthetic glucocorticoids (sGC) are administered to women at risk for pre-term delivery to reduce respiratory distress syndrome in the newborn. The prefrontal cortex (PFC) is important in regulating stress responses and related behaviours and expresses high levels of glucocorticoid receptors (GR). Further, antenatal exposure to sGC results in a hyperactive phenotype in first generation (F_1_) juvenile male and female offspring, as well as F_2_ and F_3_ juvenile females from the paternal lineage. We hypothesized that multiple courses of antenatal sGC modify gene expression in the PFC, that these effects are sex-specific and maintained across multiple generations, and that the gene sets affected relate to modified locomotor activity. We performed RNA sequencing on PFC of F_1_ juvenile males and females, as well as F_2_ and F_3_ juvenile females from the paternal lineage and used regression modelling to relate gene expression and behavior. Antenatal sGC resulted in sex-specific and generation-specific changes in gene expression. Further, the expression of 4 genes (*C9orf116*, *Calb1*, *Glra3*, and *Gpr52*) explained 20–29% of the observed variability in locomotor activity. Antenatal exposure to sGC profoundly influences the developing PFC; effects are evident across multiple generations and may drive altered behavioural phenotypes.

## Introduction

The prefrontal cortex (PFC) is essential for top-down regulation of neuroendocrine and behavioural processes^1,2^. Glutamatergic efferents project from the PFC to forebrain regions that then project GABAergic efferents to the paraventricular nucleus (PVN), decreasing the hypothalamic-pituitary-adrenal (HPA) axis response to stress^2,3^. The PFC is also highly sensitive to environmental stimuli (e.g. stress, sleep, diet), in particular, stimuli present during fetal and/or early postnatal life^4^. For example, antenatal exposure to high levels of glucocorticoids (GCs) programs changes in gene expression in the PFC that persist through adulthood^5^. Furthermore, altered signaling of key pathways in the PFC, such as the GABAergic signaling pathway, have been implicated in many psychiatric disorders that have developmental origins, including Attention Deficit Hyperactivity Disorder (ADHD)^6^, posttraumatic stress disorder (PTSD), major depressive disorder (MDD), and bipolar personality disorder (BPD)^7^. Thus, the PFC is a critical brain region of interest to the study of the impact of fetal exposures.

Antenatal synthetic glucocorticoids (sGC) are administered to women at risk for preterm delivery to decrease the morbidity and mortality in the newborn associated with preterm birth (e.g. respiratory distress syndrome)^8–10^. This life-saving treatment has also been associated with an increased risk of developing stress-related behavioural problems, including anxiety, hyperactivity, and distractibility in children born preterm and children born at term; female children are affected more than male children^11^. sGCs exert their effects by primarily binding to glucocorticoid receptors (GR), which translocate to the nucleus and bind glucocorticoid response element (GRE) regions in the DNA to regulate gene expression^12,13^. The GR is highly expressed in the developing prenatal brain, especially in the PFC^14^. Antenatal sGC exposure alters the expression of genes related to ADHD in the prefrontal cortex of marmoset monkeys^5^, and affects the volume of brain regions involved in regulating behaviour in human infants^15^ and children^16^. In animal studies, we have previously demonstrated that antenatal exposure to sGC results in widespread changes in gene expression in the fetal brain^17^. Further, the effects of sGC exposure on gene expression and behaviour transmit across multiple generations of juvenile offspring in the guinea pig in a sex-specific manner^18^. The strongest effects of sGC, a hyperactive phenotype in an open-field environment, were observed in F_1_ males and three generations of juvenile female offspring from the paternal lineage^18^. Guinea pigs were selected for these studies as this species exhibits similar profiles of fetal neurodevelopment and placentation to the human, in addition to having a long gestation (approximately 69 days), which allows targeting of antenatal treatments to specific phases of development^19,20^.

Understanding the relationship between patterns of gene expression and phenotype provides greater insight into the molecular mechanisms that are affected by prenatal sGC exposure. Here, we investigate the effects of antenatal sGC on the transcriptome in the guinea pig PFC in those animals that displayed increased locomotor activity in the open-field. We hypothesize that the antenatal exposure to sGC programs changes in gene expression patterns in the PFC of three generations of juvenile female guinea pig offspring and first generation juvenile males, and that the effects of sGC exposure on gene expression are associated with the hyperactive locomotor behaviour observed in these animals.

## Results

Animals used for molecular analysis in the present study, were a subset of those where behavioural data (including open-field activity) were presented in a previous publication^18^. Samples were drawn based on RNA availability and quality.

### Gene Expression: Females

**Figure 1:**
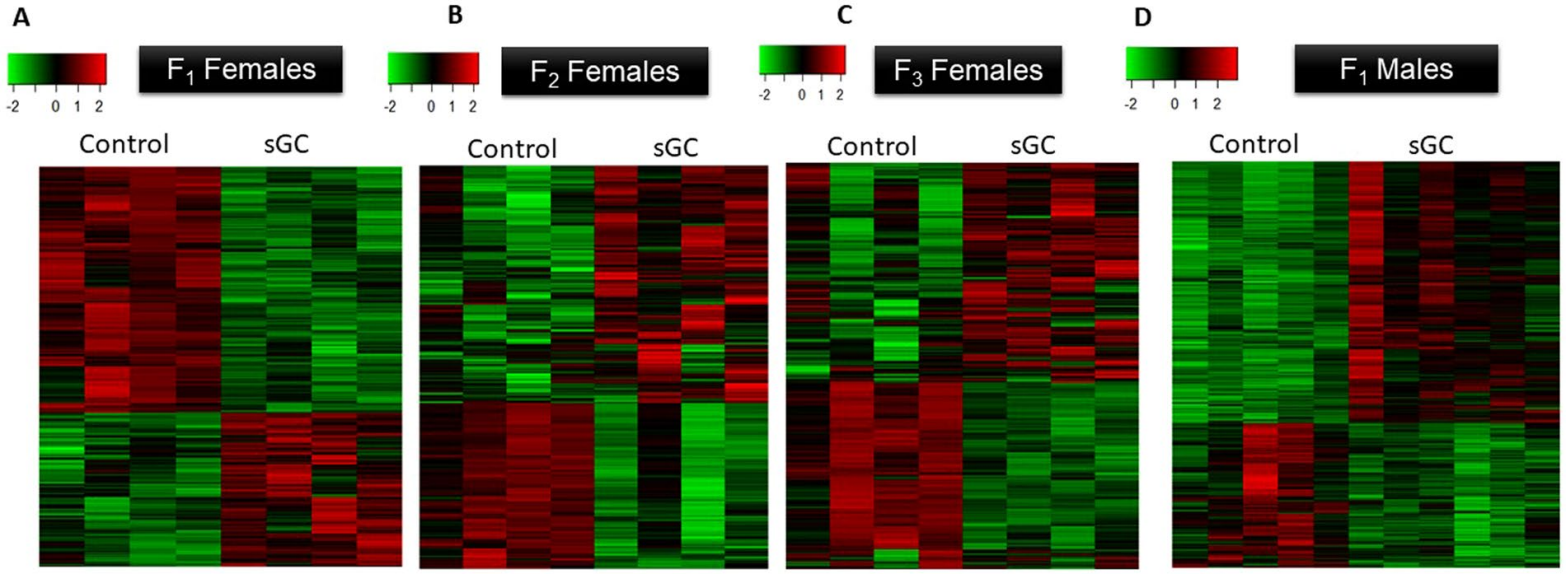
Heat maps of significantly differentially expressed genes in F_1_- F_3_ juvenile female offspring and F_1_ juvenile male offspring (P < 0.001, FDR < 0.05). Each row represents one gene, each column represents one animal. The genes are plotted by Euclidean distance using the complete clustering method. The colours in the heatmap display the gene expression relative to the two groups. A gene with higher counts relative to other samples, is indicated in red, a gene with lower counts is indicated in green. (**A**) In F_1_ juvenile female sGC offspring, 1148 genes were significantly (P < 0.001, FDR < 0.05) differentially expressed relative to Control. Of these, 442 genes were significantly up-regulated, and 706 genes down-regulated. (**B**) In F_2_, 432 genes were significantly (P < 0.001, FDR < 0.05) differentially expressed between Control and sGC, with 255 genes up-regulated and 177 genes down-regulated. (**C**) In F_3_, 438 genes were significantly (P < 0.001, FDR < 0.05) differentially expressed following prenatal sGC. Of these, 258 genes were significantly up-regulated and 180 genes down-regulated. (**D**) In the F_1_ juvenile sGC males, a total of 996 genes were significantly (P < 0.001, FDR < 0.05) differentially expressed. Of those, 354 genes were downregulated, and 642 genes were significantly upregulated.

**Figure 2:**
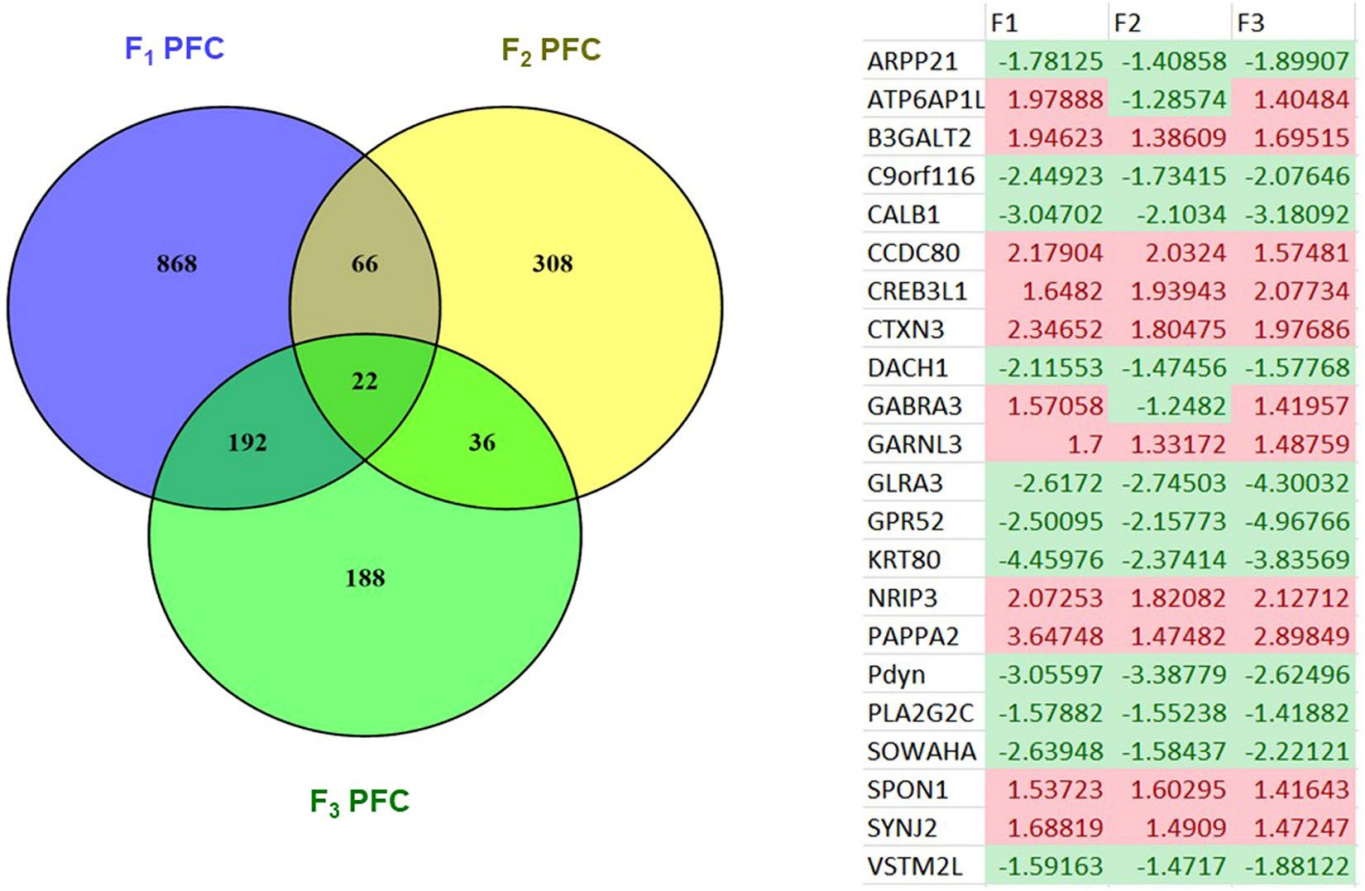
(**A**) Venn diagram illustrating the number of genes that were significantly differentially expressed (P < 0.001, FDR < 0.05) in the PFC from F_1_-F_3_ sGC females and the number of genes that overlap between generations. (**B**) Expression changes of the 22 genes that were differentially expressed (P < 0.001, FDR < 0.05) in all three generations of female offspring. Values indicate the fold-change in gene expression in sGC animals relative to control, colour further indicates the direction of change (green: significantly down-regulated, red: significantly upregulated).

In F_1_ female offspring, 1148 genes were significantly differentially expressed in the PFC of animals born to sGC treated mothers compared to controls (Fig. 1A; FDR < 0.05). Of these, 442 genes were significantly up-regulated and 706 genes down-regulated. GSEA for differentially expressed genes between sGC and control groups revealed enrichment of 322 gene sets (Supplementary Table 1; NES > 1.6, FDR < 0.25); 91 gene sets were positively enriched (i.e. increased expression in sGC vs. control) and 231 gene sets were negatively enriched (i.e. decreased expression in sGC vs. control). In F_2_ offspring, 432 genes were significantly differentially expressed between the sGC and control groups (Fig. 1B; FDR < 0.05), with 255 genes up-regulated and 177 genes down-regulated. GSEA revealed 56 enriched gene sets (Supplementary Table 1; NES > 1.6, FDR < 0.25); 53 were positively enriched and 3 negatively enriched; NES > 1.6, FDR < 0.25). In F_3_ offspring, 438 genes were significantly differentially expressed in the sGC group compared to controls (Fig. 1C; P < 0.001, FDR < 0.05), 258 genes were significantly up-regulated and 180 genes down-regulated; NES > 1.6, FDR < 0.25. GSEA identified 162 enriched gene sets, with 116 positively enriched and 46 negatively enriched (Supplementary Table 1; NES > 1.6, FDR < 0.25). There were 22 genes that significantly differentially expressed in all three generations of female offspring (Fig. 2, FDR < 0.05).

### Gene Expression: Males

In the F_1_ juvenile males, a total of 996 genes were significantly differentially expressed in sGC offspring relative to control (Fig. 1D; FDR < 0.05). Of the differentially expressed genes, 354 were significantly down-regulated and 642 genes were significantly up-regulated. GSEA identified 157 gene sets that were significantly enriched in F_1_ sGC male offspring compared to controls. 48 gene sets were negatively enriched in the F_1_ sGC male offspring, while 109 pathways were positively enriched (Supplementary Table 1; NES > 1.6, FDR < 0.25).

### Gene Expression: Female vs. Male Comparisons

**Figure 3:**
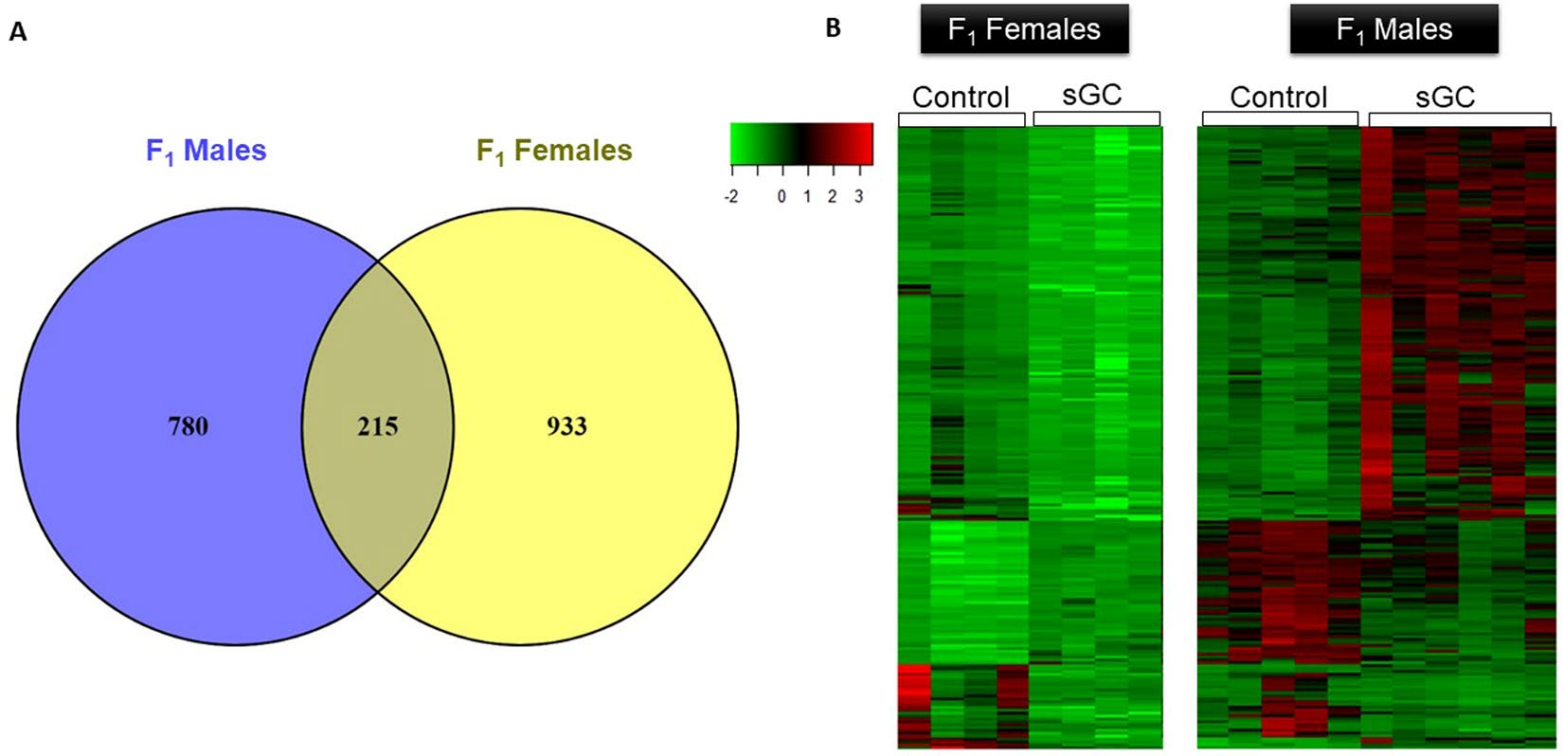
(**A**) Venn diagram illustrating the number of genes that were significantly differentially expressed in the PFC from F_1_ female and F_1_ male sGC offspring and the number of genes that overlap between generations. (**B**) Heatmap of the 215 genes that were differentially expressed in F_1_ female and F_1_ male sGC offspring (P < 0.001, FDR < 0.05). Each row represents one gene, each column represents one animal. Green represents low expression and red represents high expression.

There were 215 genes that were significantly differentially expressed in the PFC from F_1_ sGC female and male offspring (FDR < 0.05; Supplementary Table 2**;** Fig. 3A). There were 22 genes that were down-regulated in both male and female offspring whose mothers had been exposed to sGC (Supplementary Table 2**)** and were shown to be significantly enriched for the locomotor behavior pathway by ConsensusPathDB (p < 0.001, FDR < 0.05). The expression of the remaining 193 genes was divergent in males and females (i.e. up in males and down in females, or vice-versa; Fig. 3B). GSEA showed 51 gene sets were enriched in both sGC female and male offspring, however the enrichment occurred in opposite directions in each sex (i.e. increased in males and down in females, or *vice-versa*; NES > 1.6, FDR < 0.25; Supplementary Table 3**)**. Since the sGC offspring in all four groups of sGC animals (F_1_-F_3_ Females and F_1_ males) displayed increased open-field activity, we investigated common genes that were differentially expressed in all four groups. The hypothesis was that despite sex-specific changes in gene expression, there may be genes common to all groups that are associated with the observed open-field activity in these animals. Ten genes: *Arpp21*, *Atp6ap1l*, *C9orf116*, *Calb1*, *Glra3*, *Gpr52*, *Krt80*, *Pdyn*, *Sowaha*, *Vstm2l*, were differentially expressed in all four groups of sGC animals (FDR < 0.05; F_1_-F_3_ Females and F_1_ males; Table 1).

**Table 1:** Expression changes of the 10 genes that are differentially expressed in all four groups of sGC offspring.

Gene	F_1_ Males	F_1_ Females	F_2_ Females	F_3_ Females
*Arpp21*	−1.27	−1.78	−1.41	−1.90
*Atp6ap1l*	−1.18	1.98	−1.29	1.40
*C9orf116*	−1.47	−2.45	−1.73	−2.08
*Calb1*	−1.96	−3.05	−2.10	−3.18
*Glra3*	−3.73	−2.62	−2.75	−4.30
*Gpr52*	−2.28	−2.50	−2.16	−4.97
*Krt80*	−2.78	−4.46	−2.37	−3.84
*Pdyn*	−3.08	−3.06	−3.39	−2.62
*Sowaha*	−1.51	−2.64	−1.58	−2.22
*Vstm2l*	−1.62	−1.59	−1.47	−1.88

Values indicate the fold-change in gene expression in sGC animals relative to control. Positive numbers indicate significantly upregulated expression, negative numbers indicate significantly down-regulated expression.

### Regression Results

**Figure 4:**
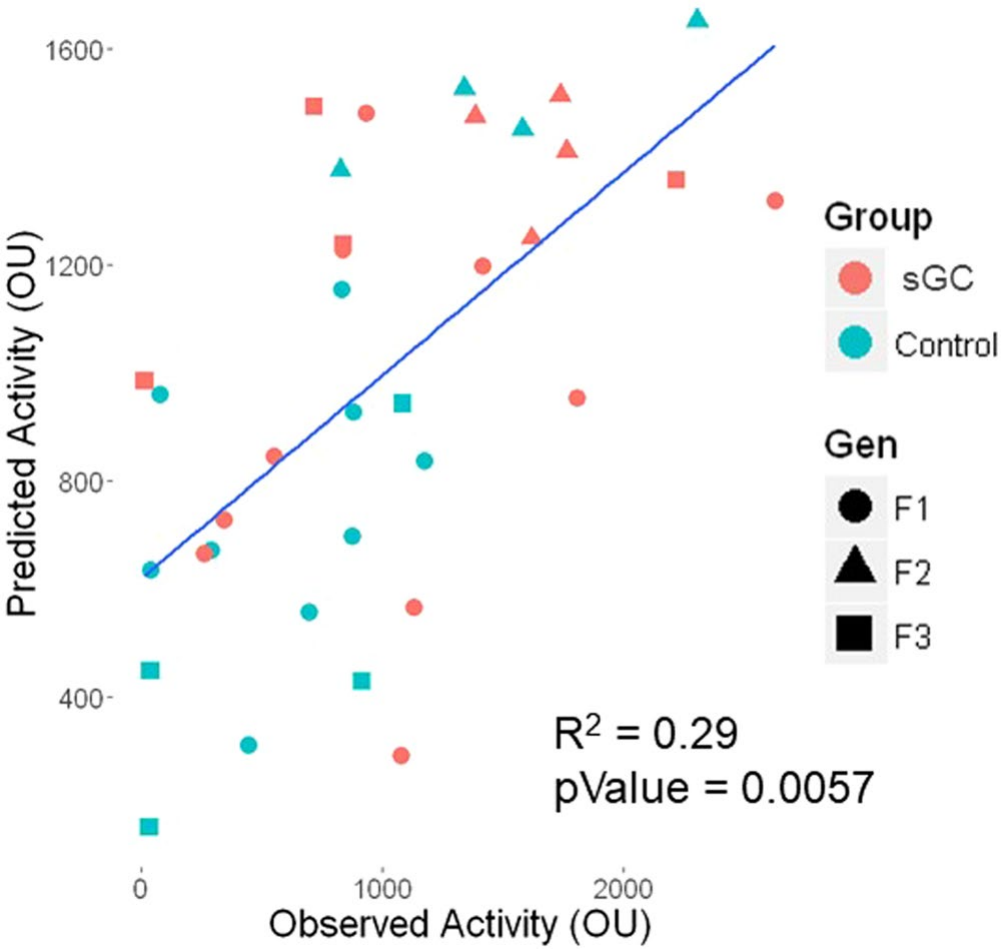
Linear regression of activity predicted from the expression of *C9orf116*, *Calb1*, *Glra3* and *Gpr52* (Predicted Activity (OU)) over experimentally observed activity (Observed Activity (OU)) adjusted R^2^ = 0.29, p-value = 0.0057.

Recursive feature selection was used to rank the 10 genes that were differentially expressed in all four groups based on their contribution to the variation in open-field activity (Table 2). Multivariate linear regression was used to model the relationship between gene expression and behavior, with the best model being made with the inclusion of the top four genes *C9orf116*, *Calb1*, *Glra3*, and *Gpr52* from recursive feature selection (Fig. 4; adjusted R^2^ = 0.29, P = 0.006). The prediction model was validated after leave-one-out cross-validation (Supplementary Fig. 1; adjusted R^2^ = 0.20, P = 0.004).

**Table 2:** Gene ranking after recursive feature selection.

Gene	Rank
C9orf116	1
Glra3	2
Gpr52	3
Calb1	4
Krt80	5
Sowaha	6
Pdyn	7
Atp6ap1l	8
Arpp21	9
Vstm2l	10

## Discussion

Antenatal exposure to sGC resulted in changes to gene expression in the PFC that persist across three generations of juvenile female offspring derived through the paternal lineage. We previously demonstrated that F_1_-F_3_ female offspring and F_1_ male offspring display a hyperactive phenotype in the open-field test^18^. Here, we observed striking sex-specific effects of sGC on gene transcription in the PFC, with a small overlap (~10%) in the number of genes that were affected by sGC in F_1_ females and males. While 193 genes were differentially expressed in opposite directions, there were 22 genes that were down regulated in both male and female sGC F_1_ offspring, and these genes were enriched for locomotor activity. Furthermore, we identified four differentially expressed genes in F_1_-F_3_ female offspring and F_1_ male offspring that were associated with 20–29% of the open-field activity variability, thereby providing insight into changes in gene expression following sGC that may mediate behavioural outcomes in both male and female offspring.

### Transgenerational Effects of Antenatal sGC and GABAergic Expression Pathways

In F_1_ offspring, the expression of GABAergic signaling genes (*Gabra3a*, *Gad2*)^21^ were significantly altered in the PFC of animals exposed to antenatal sGC. *Gad2*, that encodes glutamic acid decarboxylase was significantly down-regulated in the sGC females, while *Gabra3a*, the primary GABA receptor in PFC neurons, was significantly up-regulated. These changes may indicate that F_1_ female offspring of sGC-treated mothers had decreased GABA neurotransmitter levels, which has previously been shown to result in increased HPA function^22^ and a hyperactive phenotype^23^. Furthermore, the expression of GABAergic signaling genes, *Gabra2* and *Gabra3a* were significantly down-regulated in F_2_ sGC offspring, while *Gabra1* and *Gabra3* were significantly upregulated, with *Gad2* significantly down regulated in F_3_ sGC animals. These data indicate that altered gene expression related to GABAergic signaling persists over multiple generations. Altered GABAergic signaling in the PFC has been previously observed in patients with schizophrenia, bipolar disorder, and major depressive disorder^21,24^, and early life exposure to sGC has been linked to development of psychiatric disease^25^. Therefore, the changes in expression for GABAergic genes that we observe following exposure to sGC may be associated with increased risk of psychiatric disease later in life.

### Sex-Specific Effects of Antenatal sGC on PFC Gene Expression: Open-Field Activity

Prenatal exposure to sGC resulted in substantial changes in gene expression in the PFC that extended, at least, up to 50 days after exposure in F_1_ male and female offspring. Consistent with previous literature, we observed sex- and generation-specific programming following antenatal sGC exposure^18,26^. All 51 commonly enriched gene sets were affected in the opposite direction in male and female offspring. The gene sets most affected included extracellular ligand-gated ion channel activity (critical for intercellular communication^27^) and synaptic signaling (synapse formation^28^). Both pathways were up-regulated in females, and down-regulated in males. These pathways play a pivotal role in information processing allowing appropriate behavioural responses and adaptation^27,28^. Since these pathways were enriched in opposite directions in males and females, it is possible that simply perturbing these pathways is sufficient to produce a hyperactive phenotype. Conversely, enrichment of these pathways may not play a significant role in the observed hyperactive phenotype^18^, and further detailed investigation is required.

Greater insight regarding the relationship between gene expression and behaviour may come from the genes that were significantly differentially expressed in both male and female sGC offspring. Of the 215 genes that were differentially expressed in both male and female offspring, 193 were expressed in opposite directions, but there were 22 genes that were significantly down-regulated in both sGC male and female F_1_ offspring, and these genes were enriched for the locomotor behaviour pathway. These findings suggest that despite the major sex-specific differences in gene expression, the hyperactive phenotype observed in both males and females may be mediated by the same transcriptional pathways in both sexes.

### Transgenerational Effects of Antenatal sGC: Molecular and Behavioural Correlations

Since all three generations of sGC females and F_1_ males displayed increased open field activity, we investigated changes in gene expression that occurred in all four groups of sGC offspring to identify genes related to the behavioural phenotype. There were 10 genes (*Arpp21*, *Atp6ap1l*, *C9orf116*, *Calb1*, *Glra3*, *Gpr52*, *Krt80*, *Pdyn*, *Sowaha*, *Vstm2l*) significantly differentially expressed in all three generations of female offspring and in the F_1_ males. It is important to note that the expression of these 10 genes was not altered in the PVN of the same female offspring following antenatal sGC exposure^18^, indicating region-specific effects. Feature selection analysis and multivariate linear regression analysis suggest that the expression of four of these genes, Chromosome 9 Open Reading Frame 116 (*C9orf116*), Calbindin 1 (*Calb1*), Glycine Receptor Alpha 3 (*Glra3*), and G Protein-Coupled Receptor 52 (*Gpr52*), are involved in the hyperactive behavioural phenotype observed in the sGC-exposed offspring lineage. The expression of these genes was significantly decreased in all four groups of sGC animals. While these genes have not been previously studied in the context of antenatal sGC exposure and locomotor activity, each gene plays an essential role in processes that are integral to governing locomotor behaviour.

*C9orf116* expression is directly regulated by p53, and *C9orf116* knockdown down-regulates proapoptotic genes, implicating a role in apoptosis^29^. Reduced expression of genes involved in apoptosis has previously been observed in isolation-reared rats that displayed a hyperactive phenotype in the open-field, and may be related to changes in apoptotic levels that alter neural plasticity in the PFC^30^.

Calb1 is a high-affinity calcium buffer/sensor in pyramidal, nonpyramidal, and GABAergic interneurons in the PFC^31^. *Calb1* has a protective effect against neuronal injury from excess Ca^2+^ exposure^32^. *Calb1* is regulated by estrogen and androgens, creating sex-specific differences in its expression^31^. Antenatal sGC exposure decreases *Calb1* expression in the basolateral amygdala^33^ and *Calb1* expression is decreased in rats weaned in isolation, resulting in decreased exploratory behaviour^34^. *Calb1* knock-out animals display decreased expression of GABAergic signaling genes (previously linked to hyperactive phenotype^23^), which is consistent with the changes observed in the sGC offspring in the present study. Therefore, *Calb1* expression has been shown to be affected by antenatal sGC and altered expression has been shown to influence open-field activity. The observed decrease in *Calb1* expression in the sGC offspring may influence open-field activity through GABAergic interactions.

Gpr52 is an orphan g-protein coupled receptor that is expressed exclusively in the brain^35^. *Gpr52* knock-out has anxiolytic effects on behaviour in mice^36^. In humans, *GPR52* expression profiles overlap with the distribution of D1 dopamine receptors in the PFC, and it is thought that the expression of Gpr52 influences locomotor activity through activation of the dopamine receptor D1 (DRD1) and N-methyl-D-aspartate (NMDA) receptors in the PFC through intracellular cAMP accumulation^36,37^. Of note, *Drd1* expression is significantly down-regulated in F_1_ sGC females, and significantly upregulated in F_2_ sGC females, while expression of *Grin2a*, which encodes for the NMDA receptor, is significantly upregulated in F_3_ sGC females, which may present a plausible mechanism by which the decreased *Gpr52* expression observed in the sGC offspring influences open-field activity.

Glycine receptors, such as Glra3 play a fundamental role in mediating inhibitory neurotransmission throughout the central nervous system^38^. Glycine receptor knock-out animals show increased locomotor activity in the open-field when stimulated with low levels of ethanol^39^. This may occur due to neuronal disinhibition from reduced effects of ethanol on glycine receptors^39^. The decreased *Glra3* expression in the sGC animals may increase neuronal disinhibition, and play a role in the increased open-field activity observed in the sGC-exposed offspring lineage^18^.

The reduced expression of these four genes, selected from recursive feature selection analyses, explained between 20–29% of the variability in hyperactive behaviour observed in F_1_ males and F_1_-F_3_ juvenile female offspring. While altered expression of these genes has previously been shown to influence locomotor activity, future experiments are required to investigate the specific mechanisms by which decreased expression of *Calb1*, *Glra3*, *Gpr52*, *C9orf116* in the PFC alter open-field activity in the context of antenatal sGC exposure. Though changes in gene expression in the PFC can provide some insight into the sources of variability contributing to increased open-field activity, 70–80% of the variability remains to be explained. The PFC has glutamatergic efferents that directly connect to the ventral tegmental area (VTA) and the nucleus accumbens (Nac), which have been connected to locomotor activity^40^. It is possible that dysregulated gene expression in the PFC has downstream effects in other brain regions that contribute to the hyperactive phenotype observed in the sGC offspring and merit further investigation. It is also possible that gene expression changes in the PFC and behaviours are independent and may be a result of parallel downstream effects of sGC, though given the pivotal role that the PFC plays in behaviour, this would appear unlikely.

These findings demonstrate paternal transmission of the effects of antenatal sGC over three generations of female offspring, yet the mechanism of transmission has yet to be elucidated. We have shown that antenatal sGC exposure results in a complex pattern of effects that are dynamic and dependent on sex, age, generation, brain region, and parental line of transmission^18^, which is consistent with other instances of transgenerational transmission^41^. Unique to the present study is the identification of select genes that are consistently altered across all four groups of sGC offspring and relate to a hyperactive phenotype. These findings may indicate that PFC signaling plays a critical role in propagating the effects of antenatal sGC.

## Conclusion

We have demonstrated transgenerational changes in gene expression that relate to the behavioural phenotypes observed in the juvenile offspring. Antenatal exposure to sGC resulted in a pattern of gene expression in the PFC consistent with reduced GABAergic signaling in F_1_-F_3_ offspring. As disruption of GABAergic signaling is common in major psychiatric diseases, and as sGC exposure is associated with increased risk for developing psychiatric disease^25^, this pattern of gene expression may provide a mechanism by which antenatal sGC exposure contributes to psychiatric vulnerability. Despite observing major sex- and generation-specific differences in the effects of sGC on gene expression, we identified four genes that may contribute to 20–29% of the variability in locomotor activity in F_1_ sGC males and all three generations of sGC female offspring. These findings demonstrate that multiple courses of antenatal sGC result in permanent changes in gene expression that likely alter phenotype over three generations. Follow-up studies in human cohorts are imperative to ascertain the long-term effects of sGC on neural development.

## Materials and Methods

### Animals

Pregnant guinea pigs received 3 courses of the sGC betamethasone (sGC; 1 mg/kg) or saline control in late gestation, as previously described^18^. The dose of sGC used is comparable to that administered to pregnant women at risk of preterm delivery (~0.25 mg/kg) as the glucocorticoid receptor (GR) in guinea pigs has a 4-fold lower affinity for sGC^42^. First (F_1_) and second (F_2_) generation male offspring were mated with non-experimental females to generate F_2_ and F_3_ offspring, as previously described^18^. Total locomotor activity in the open-field test (open-field activity; OFA) was measured in female and male offspring on postnatal day 19, and brains were collected at day 40, as previously reported^18^. The locomotor activity in the open-field, of the animals used for molecular analysis in the present study, was presented previously^18^. The right frontal cortex from the F_1_ males and F_1_-F_3_ paternal line females were cryosectioned at −20 °C. 1.0 mm diameter punches (Harvard Apparatus Inc., Holliston, MA, USA) of the mPFC cingulate cortex area 1 and infralimbic cortex were taken from F_1_ (Control; n = 4, sGC; n = 4), F_2_ (Control; n = 4, sGC; n = 4), and F_3_ (Control; n = 4, sGC; n = 4) females and F_1_ males (Control; n = 5, sGC; n = 6) as previously reported^18^. Only one animal of each sex from each litter was used in the molecular analysis of female offspring. Animals for RNA-seq analysis were selected based on the availability of sufficient high-quality RNA. All protocols were approved by the Animal Care Committee at the University of Toronto in accordance with the Canadian Council on Animal Care.

### RNA Sequencing

RNA was extracted from punches using the AllPrep Universal Kit (Qiagen, Ontario, Canada) and RNA quality was determined by Bioanalyzer (RNA 6000 Pico LabChip, Applied Biosystems, Ontario, Canada); all RNA samples RIN ≥ 7. mRNA library preparation was performed using Illumina TruSeq V2 mRNA enrichment using standard protocols. High-throughput sequencing were performed on an Illumina HiSeq2500 sequencing system using standard run, following the protocol recommended by Illumina for sequencing mRNA samples. Sequencing was undertaken for each biological replicate at 1 × 51 bp (Donnelly Centre for Cellular and Biomolecular Research; Toronto, Canada). RNA-seq results were analyzed, as previously described^18^. Briefly, differential gene expression was assessed using EdgeR’s (version 3.12.1)^43,44^ general linear model likelihood ratio test and FDR-corrected *p* < 0.05 was considered significant. qPCR validation correlated 93% with RNAseq findings (Supplementary Fig. 2). Genotype permutations (1000) were computed in Broad Institute’s Gene Set Enrichment Analysis (GSEA)^45,46^ to determine FDR, nominal p- value, and normalized enrichment score (NES) of each gene set. Gene sets with FDR ≤ 0.25, p ≤ 0.01, and NES ≥ 1.6 met significance thresholds^18^. While GSEA provides insight into how the expression of genes from an individual pathway are altered, over-representation analysis indicates whether there are more genes in a set that are related to an individual pathway than would be expected by chance. ConsensusPathDB was used to perform over-representation analysis of significantly differentially expressed genes^47^; enrichment with a p-value < 0.001, FDR < 0.05 was considered significant. qRT-PCR validations were run using cDNA that was made by SensiFAST cDNA synthesis kit (Bioline, London, England). qRT-PCR was run in triplicate (SensiFAST SYBER Hi-ROX 20 μl reaction, Bioline) and quantified by a CFX96 Real-Time System (Bio-Rad). Expression of target mRNA (Supplementary Table 4) relative to *Gapdh* housekeeping gene was assessed using the 2^−ΔΔct^ method.

### Behavioural and Molecular Correlations

To identify genes that are associated with locomotor activity, recursive feature selection^48^ was performed on the normalized gene expression counts for the genes that were significantly differentially expressed in F_1_ sGC males, and in all 3 generations of sGC female animals. First, the expression of all the genes were fitted in a linear regression to predict open field activity. The coefficients of each gene were used to rank the genes from highest contribution to open field activity to lowest. The gene with the lowest contribution to open-field activity was removed, and the remaining genes were fitted in a new linear regression to predict open-field activity. This process was repeated until all the genes were ranked in order of contribution (or importance) to open-field activity^48^. The expression values for the top four feature selected genes (*C9orf116*, *Calb1*, *Glra3*, and *Gpr52*) were input into a multiple regression to predict open-field activity, and the coefficient of determination was calculated. The model was validated using leave-one-out cross validation.

## Supplementary information

Supplementary Information

Supplementary Table 1

Supplementary Table 3

## Data Availability

All sequencing data can be found under GEO submission ID: GSE107415. Computer code available upon request.
