# Supplementary material for: Antenatal Glucocorticoid Exposure Results in Sex-Specific and Transgenerational Changes in Prefrontal Cortex Gene Transcription that Relate to Behavioural Outcomes
Source: Sci Rep. 2019 Jan 24;9:764. doi: 10.1038/s41598-018-37088-3 (PMC6346022; doi:10.1038/s41598-018-37088-3)
Supplement: Supplementary file 1 — Supplementary Information [file 41598_2018_37088_MOESM1_ESM.pdf]

Supplementary Information

Antenatal glucocorticoid exposure results in sex-specific and transgenerational changes in prefrontal cortex gene transcription that relate to behavioural outcomes

Constantinof A, Moisiadis VG, Kostaki A, Szyf M, Matthews SG

Supplementary Figures

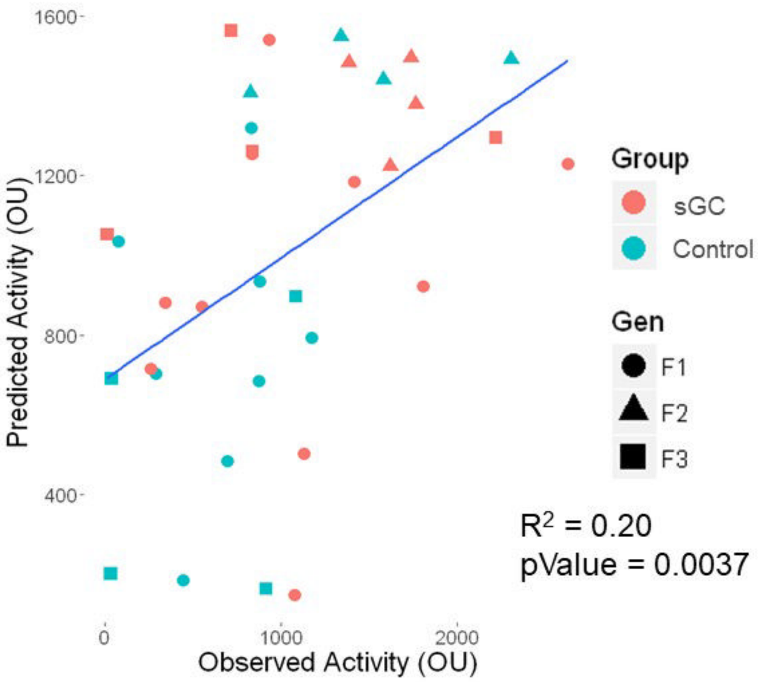

Supplementary Figure 1. Linear regression of activity predicted from the expression of *C9orf116, Calb1, Glra3* and *Gpr52* using leave-one-out cross-validation (Predicted Activity (OU)) over experimentally observed activity (Observed Activity (OU)) adjusted R2= 0.20, pvalue = 0.0037.

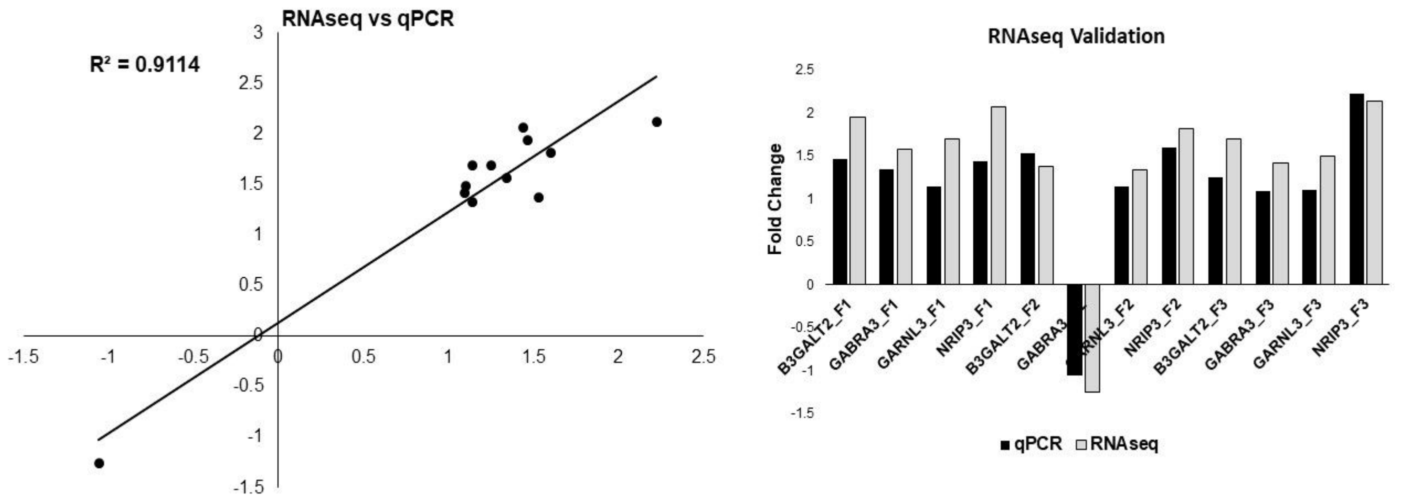

Supplementary Figure 2. qRT-PCR validation of RNA-seq results. A) Correlation of sequencing data to qPCR data (each point represents one gene): R2=0.9114. B) Comparison of fold-change (from control) in expression observed from RNA-seq (grey bars) and qPCR (solid bars).

Supplementary Tables

Supplementary Table 2: Expression changes of the 215 genes that are differentially expressed in F1 male and female offspring. Values indicate the fold change in gene expression in sGC animals relative to control, color further indicates the direction of change (green: significantly down-regulated, red: significantly upregulated).

Gene	F_1 Females	F_1 Males
MARCH4	-1.49	-1.42
ANKRD63	-2.61	-1.34
ARPP21	-1.78	-1.27
C9orf116	-2.45	-1.47
CALB1	-3.05	-1.96
CAMK1G	-1.52	-1.22
EDAR	-1.64	-1.87
ESR2	-1.94	-1.45
GLRA3	-2.62	-3.73
GPR52	-2.50	-2.28
KRT80	-4.46	-2.78
MCOLN3	-2.65	-2.02
MESDC1	-1.91	-1.25
NECAB2	-1.94	-1.82
NHS	-1.47	-1.32
PBX3	-1.49	-1.45
Pdyn	-3.06	-3.08
PLPPR1	-1.92	-1.37
SOWAHA	-2.64	-1.51
SYTL5	-3.43	-1.57
VSTM2L	-1.59	-1.62
ZBTB7C	-1.68	-1.40
ABHD3	-1.49	1.48
ACKR3	-1.67	1.38
ACSF2	-1.41	1.21
ADAM17	-1.43	1.46
ADAMTS4	-1.90	1.93
ALDH7A1	-1.37	1.15
APRT	-1.34	1.20
ARHGEF37	-1.53	1.59
BBOX1	-1.76	1.48
BCHE	-1.69	1.47
BGN	-1.54	1.29
BMP1	-1.65	1.21

**C3orf70**	-1.49	1.24
**CA5B**	-1.47	1.32
**CARHSP1**	-2.49	1.19
**Cat**	-1.43	1.15
**CCDC153**	-1.88	1.76
**CCDC88C**	-2.33	1.30
**CD48**	-1.64	1.37
**CD74**	-1.33	1.28
**CD82**	-1.65	1.47
**CDC42EP2**	-1.73	1.19
**CDK2AP2**	-1.55	1.21
**CDK5RAP2**	-1.53	1.45
**CFD**	-1.81	1.32
**CHN2**	-2.32	1.28
**CHST3**	-1.64	1.29
**CHST4**	-2.00	1.69
**CLIC4**	-1.48	1.42
**CMTM5**	-1.54	1.56
**CNN2**	-1.54	1.27
**COL5A3**	-3.03	1.98
**COL9A3**	-1.63	1.27
**CREB3L2**	-1.50	1.43
**CTDSP1**	-1.44	1.27
**CTDSP2**	-1.35	1.16
**CTNNA1**	-1.36	1.29
**DHRS3**	-1.73	1.28
**DOCK10**	-1.72	1.62
**DOCK2**	-1.60	1.66
**ECEL1**	-2.28	1.35
**EDNRB**	-2.03	1.37
**EFS**	-1.60	1.47
**ELOVL5**	-1.32	1.25
**FADS2**	-1.30	1.17
**FAM173A**	-1.57	1.30
**FAM189A2**	-1.70	1.41
**FAM222A**	-1.94	1.42
**FAM53B**	-1.57	1.40
**FEN1**	-1.40	1.14
**FLNB**	-1.52	1.21
**FRMD8**	-1.63	1.37
**GFAP**	-2.19	1.66
**GNG7**	-3.03	1.43
**GPAM**	-1.32	1.18

**GPR17**	-1.54	1.24
**GPR37L1**	-1.41	1.37
**GPR84**	-1.64	1.42
**GPRC5B**	-1.50	1.38
**GPRC5C**	-1.71	1.61
**HDAC1**	-1.44	1.21
**HEPACAM**	-1.43	1.36
**HMGB2**	-2.06	1.39
**HN1L**	-1.51	1.46
**HPS5**	-1.55	1.35
**IGFBP7**	-1.35	1.22
**INPPL1**	-1.49	1.22
**IQCK**	-1.46	1.37
**ITGB2**	-1.53	1.33
**JOSD2**	-1.47	1.40
**KANK1**	-1.84	1.43
**KAT2B**	-1.68	1.31
**KCNJ10**	-1.40	1.28
**KIAA1755**	-2.07	1.83
**KIF1C**	-1.46	1.33
**LAMA2**	-2.31	1.41
**LAPTM5**	-1.44	1.20
**LGALS1**	-1.85	1.69
**LIMS2**	-1.54	1.24
**LIPE**	-1.78	1.89
**LIPK**	-1.77	1.97
**LPAR1**	-1.67	1.58
**LRP10**	-1.53	1.34
**LRRC1**	-1.90	1.63
**MARCKSL1**	-1.43	1.21
**MEGF10**	-1.38	1.20
**MOB3B**	-1.66	1.45
**MOG**	-1.84	2.00
**MSTN**	-2.01	1.75
**NFE2L2**	-1.41	1.31
**NKX2-2**	-1.81	1.41
**NOD2**	-1.71	1.34
**NOTCH1**	-1.47	1.25
**NPC2**	-1.38	1.20
**OLIG2**	-1.72	1.45
**P2RX7**	-1.53	1.64
**P2RY2**	-1.90	1.56
**PARVB**	-1.74	1.32

**PDK4**	-1.76	1.77
**PHGDH**	-1.44	1.22
**PHLDB1**	-1.78	1.62
**PIK3R3**	-1.40	1.26
**PLB1**	-2.24	1.89
**PLEKHA7**	-1.48	1.26
**PLEKHH2**	-1.70	1.57
**PLPP2**	-1.80	1.85
**PLXNB3**	-1.81	1.59
**PON2**	-1.42	1.25
**PRIMA1**	-2.50	1.39
**PTGDS**	-1.49	1.33
**PXN**	-1.43	1.20
**RAB7B**	-1.87	1.85
**RAPGEF3**	-1.53	1.46
**RASSF4**	-2.12	1.59
**RFTN2**	-1.60	1.26
**RGS3**	-1.68	1.50
**RHOC**	-1.53	1.28
**SASH3**	-1.55	1.26
**SFT2D2**	-1.49	1.23
**SGK2**	-2.02	1.92
**SLC25A13**	-1.67	1.27
**SOX10**	-1.69	1.70
**SOX8**	-1.65	1.36
**SREBF1**	-1.61	1.25
**SYNGR2**	-1.51	1.45
**TFEB**	-1.57	1.55
**TMCC3**	-1.55	1.36
**TMEM200B**	-4.50	2.05
**TMEM98**	-1.64	1.27
**TMPRSS5**	-2.47	1.58
**TNFAIP6**	-1.65	1.44
**TNS3**	-1.47	1.36
**TP53BP2**	-1.49	1.30
**TRPV3**	-1.99	1.91
**TSC22D4**	-1.51	1.39
**TST**	-1.89	1.29
**VAT1**	-1.47	1.32
**WASF2**	-1.40	1.23
**WFIKKN2**	-1.75	1.63
**WIPF1**	-1.78	1.52
**Ybx1**	-1.44	1.19

**ZCCHC24**	-1.51	1.46
**ZFP57**	-1.98	2.34
**ATP6AP1L**	1.98	-1.18
**ATP6V1A**	1.36	-1.17
**BPIFB6**	2.65	-1.46
**C1QL3**	2.26	-1.69
**CD247**	2.37	-1.75
**COL8A1**	2.01	-1.27
**CREG2**	1.40	-1.19
**CRIM1**	1.42	-1.20
**DEPDC1**	2.07	-1.57
**DGKG**	1.41	-1.16
**DOC2A**	1.44	-1.38
**DOK7**	1.87	-1.29
**DPP10**	1.55	-1.15
**EFR3A**	1.44	-1.21
**FAM160A1**	2.93	-1.26
**FAM81A**	1.64	-1.13
**FILIP1**	1.42	-1.34
**GPRC5A**	2.04	-1.54
**GRM2**	1.93	-1.32
**HDC**	1.49	-1.51
**HECW1**	1.82	-1.25
**Hpgd**	2.43	-1.49
**IPCEF1**	2.00	-1.24
**KIAA2022**	1.36	-1.35
**LIPM**	2.07	-1.57
**LMO4**	1.48	-1.18
**MEF2C**	1.88	-1.30
**NELL2**	1.44	-1.13
**NWD2**	1.84	-1.61
**OPCML**	1.58	-1.22
**PCSK5**	1.72	-1.68
**PGM2L1**	1.84	-1.32
**PLCH1**	1.75	-1.23
**PPFIA2**	1.41	-1.36
**PZP**	1.82	-1.57
**RAPGEFL1**	1.56	-1.16
**RBM44**	1.75	-1.43
**REPS2**	1.32	-1.19
**RIMBP2**	1.45	-1.31
**SCN2A**	1.42	-1.19
**SDR16C5**	1.93	-1.42

**SH3GL2**	1.61	-1.18
**SLA**	2.40	-1.26
**SLC39A6**	1.30	-1.17
**SMOC2**	1.70	-1.41
**SYN2**	1.61	-1.26
**SYT17**	1.61	-1.43
**THRB**	1.68	-1.23
**TRAF5**	1.41	-1.39
**XKR4**	1.68	-1.24

Supplementary Table 4: qRT-PCR Primer Pairs. Forward and reverse primer pairs used for qRT-PCR in the mPFC.

	**Forward**	**Reverse**
Garnl3	CGTGCCTGAATCCAGTCTCTT	TTTGTGGGGCTGTAGGGAAG
Gabra3	CTGGCGCTTGGTTTCTCCAG	TCCAGGGAGGATGGTCAACA
Nrip3	CTGGGCTCGTCTAAGGACAC	TCCAGCACACTGGCAAGAAA
B3galt2	TGCCAGGTAGAGCTGGATTC	TGTTTGGGGCCTCAGTGTTT
Gapdh	TGTACTGGAGGTCAATGAAGG	GTCGGAGTGAACGGATTTG
